# Impact of ambient air pollution exposure during pregnancy on adverse birth outcomes: generalized structural equation modeling approach

**DOI:** 10.1186/s12889-022-14971-3

**Published:** 2023-01-06

**Authors:** Aweke A. Mitku, Temesgen Zewotir, Delia North, Prakash Jeena, Kareshma Asharam, Sheena Muttoo, Hasheel Tularam, Rajen N. Naidoo

**Affiliations:** 1 School of Mathematics, Statistics and Computer Science, College of Agriculture Engineering and Science, University of KwaZulu-Natal, Durban, South Africa; 2 Discipline of Occupational and Environmental Health, School of Nursing and Public Health, College of Health Sciences, University of KwaZulu-Natal, Durban, South Africa; 3 Department of Statistics, Bahir Dar University, Bahir Dar, Ethiopia; 4 Discipline of Paediatrics and Child Health, School of Clinical Medicine, College of Health Sciences, University of KwaZulu-Natal, Durban, South Africa

**Keywords:** Birth cohort, Complementary log–log-link, Indirect effects, Parallel coordinates plot, Prenatal exposure

## Abstract

**Background:**

Air pollution and several prenatal factors, such as socio-demographic, behavioural, physical activity and clinical factors influence adverse birth outcomes. The study aimed to investigate the impact of ambient air pollution exposure during pregnancy adjusting prenatal risk factors on adverse birth outcomes among pregnant women in MACE birth cohort.

**Methods:**

Data for the study was obtained from the Mother and Child in the Environment (MACE) birth cohort study in Durban, South Africa from 2013 to 2017. Land use regression models were used to determine household level prenatal exposure to PM_2.5_, SO_2_ and NOx. Six hundred and fifty-six births of pregnant females were selected from public sector antenatal clinics in low socio-economic neighbourhoods. We employed a Generalised Structural Equation Model with a complementary log–log-link specification.

**Results:**

After adjustment for potential prenatal factors, the results indicated that exposure to PM_2.5_ was found to have both significant direct and indirect effects on the risk of all adverse birth outcomes. Similarly, an increased level of maternal exposure to SO_2_ during pregnancy was associated with an increased probability of being small for gestational age. Moreover, preterm birth act a mediating role in the relationship of exposure to PM_2.5_, and SO_2_ with low birthweight and SGA.

**Conclusions:**

Prenatal exposure to PM_2.5_ and SO_2_ pollution adversely affected birth outcomes after controlling for other prenatal risk factors. This suggests that local government officials have a responsibility for better control of air pollution and health care providers need to advise pregnant females about the risks of air pollution during pregnancy.

**Supplementary Information:**

The online version contains supplementary material available at 10.1186/s12889-022-14971-3.

## Background

Adverse birth outcomes are common health problems and incur significant health consequences such as infant morbidity and mortality, as well as hypertension, type 2 diabetes, and cardiovascular disease in adulthood [1–5]. Preterm birth (PB) and low birthweight [6] infants are at greater risk for mortality and a variety of health and developmental problems [7]. WHO [8] showed that in developing countries about 1 in 6 ( 16.5%) babies were born with low birthweight (< 2500 g). Globally, out of 7.6 million deaths of under-five children, 17% are due to prematurity [9]. More than 60% of preterm births (< 37 weeks gestational age) take place in South Asia and Sub-Saharan Africa [10]. Around 15% of low-birth-weight occurs in Sub-Saharan Africa [11]. Adverse birth outcomes are likely to compromise the health of the growing infant, and predict poorer health outcomes in later life [12].

Recently, there has been growing evidence that air pollution exposure plays a vital role in the occurrence of adverse pregnancy outcomes such as PB, LBW and SGA [13, 14]. Maternal exposure to air pollution during pregnancy has been suggested to be associated with increased risks of adverse birth outcomes such as PB, LBW, SGA, and intrauterine growth retardation (IUGR) [13, 15–21]. These outcomes are associated with the most commonly measured air pollutants, such as particulate matter with an aerodynamic diameter of less than 2.5 μm (PM_2.5_), sulfur dioxide (SO_2_) and oxides of nitrogen (NOx) [20]. Demographic factors [22–24], lower socio-economic status and pre-pregnancy body mass index [25] and poor housing conditions [26] are also among the risk factors for adverse birth outcomes.

In low and middle-income resource settings, alcohol use, or tobacco smoke exposure were behavioral risk factors to the health of women during their pregnancy and to that of their child [27, 28]. However, many of the studies in sub-Saharan Africa, lack the ability to adjust for these individual-level behavioural risk factors.

Generalised structural equation models (GSEM) are more appropriate than individual regression models. These models allow multiple simultaneous equations to incorporate confounding and mediation, besides incorporating latent variables for representing more complex measures that are not measurable with a single variable [29]. GSEMs minimise the effect of residual confounding in associations, especially in observational studies. GSEMs also allow for the inclusion of variables with a mediating effect on the exposure and outcome variables. In traditional regression analysis, one needs to build different models for different outcomes given a set of covariates. This makes drawing conclusions difficult and probably inaccurate. However, GSEM is applied to construct models with latent variables [30]. It encompasses unobserved external or internal variables (latent variables), along with the observed distributions [31]. Thus, a mediation analysis using GSEM was conducted to look at the direct and indirect relationships between air pollution exposure and birth outcomes.

Moreover, comprehensive models that adjust demographic, socio-economic, clinical, physical activity, and behavioural exposure predictors are needed to disentangle the impacts of prenatal air pollution exposure on adverse birth outcomes. These will promote intervention efforts to improve maternal and infant health in low and middle-income resource settings. Thus, we adopted a generalised structural equation modeling approach to address these issues and decompose the direct and indirect effects of prenatal ambient air pollution exposure on adverse birth outcomes, adjusting for prenatal exposure factors.

## Methods

### Data and variables

**Fig. 1:**
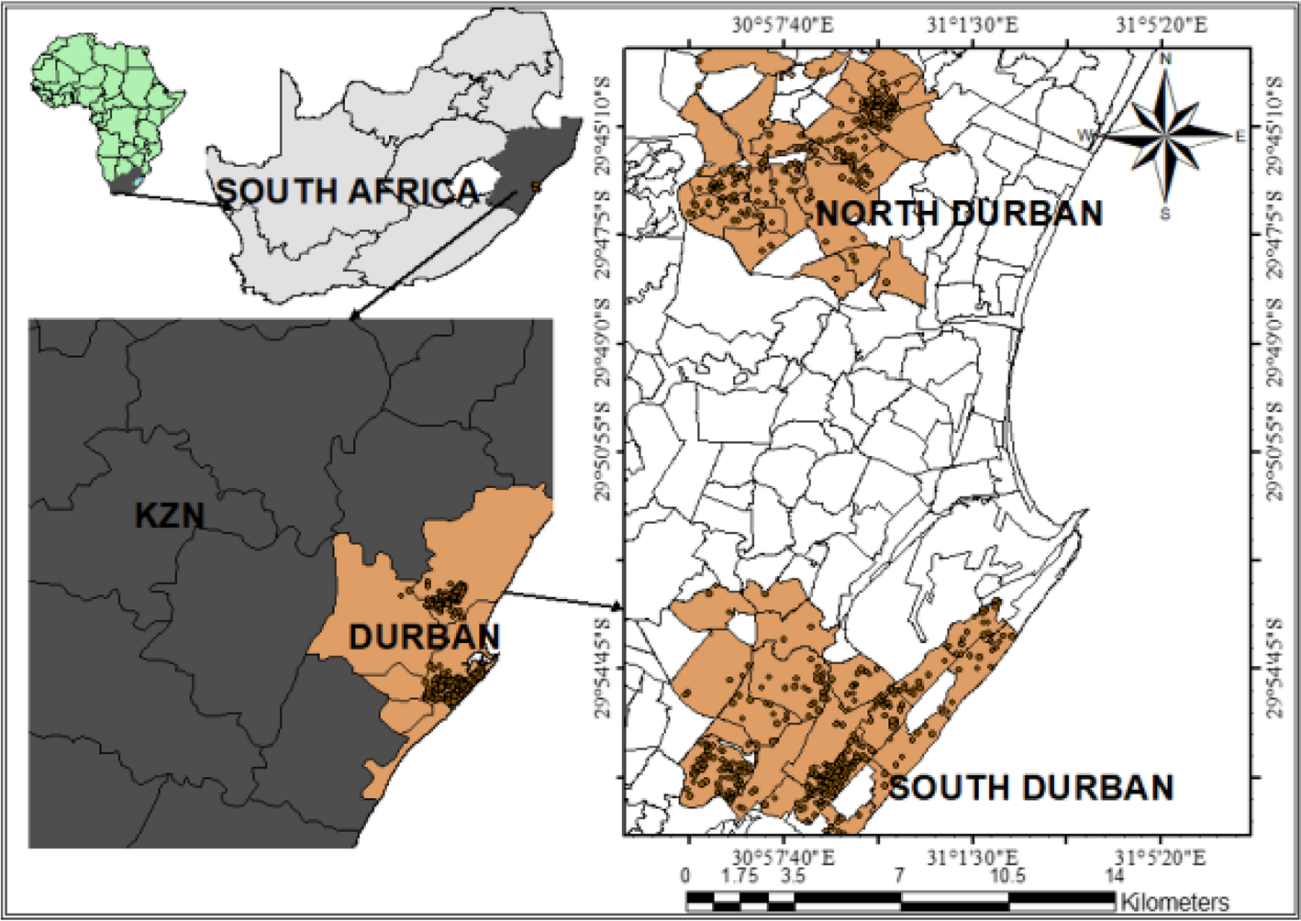
Study area of MACE birth cohort, showing the location of the study areas (north/south Durban) within the city; Dots represent study participant households from Mitku et al. [36]

Studies in the city of Durban have reported that increased levels of ambient air pollution in the city were found to be a major health concern [11, 32]. Recent studies also indicated that the participants in South Durban are exposed to high levels of NO_x_ [33, 34]. We analysed data from the Mother and Child in the Environment (MACE) birth cohort, a study with ongoing recruitment in Durban, South Africa. This is described in detail elsewhere [35]. Here we report on the enrolled cohort of 996 pregnant women from March 2013 to May 2017 from eight public sector antenatal clinics in Five communities in the south of Durban (Merebank, Bluff, Wentworth and Austerville), located in close proximity to major industries, as well as communities in less heavily industrial areas in the north of Durban (KwaMashu, and Newlands East) were selected (Fig. 1). The selected communities were similar in socio-demographic profiles.

All pregnant women that were at a gestational age of less than 20 weeks and resident for the full duration of the pregnancy in the geographical area within which the clinic was located as well as for the follow-up period of 5–6 years, were recruited into the study. Women with multiple pregnancies (*n* = 2); miscarriages (*n* = 55); stillbirths (*n* = 25); and termination of pregnancy (*n* = 2) were therefore excluded from the cohort. A further 225 participants who relocated outside the areas of interest and decide to use clinics closer to their new homes were excluded. This reduced the number of enrolled subjects followed up through to labour and delivery during their pregnancy to 687 participants. Finally, excluding 31 participants with postdate birth (gestational age above 42 weeks), the effective sample size is 656 mother–child pairs (see SupFigure 1 in Supplementary Material).

### Exposure assessment

Air pollution exposure to PM_2.5,_ SO_2_ and NOx measurements for participants in the MACE birth cohort was derived from a land use regression model. This is described in greater detail elsewhere [34, 37, 38]. Using the methodology used in the European Birth Cohorts (ESCAPE), samples of NOx were taken at 40 randomly selected sites in the north and south Durban areas (Fig. 1) using Ogawa samplers over two two-week periods during mid-summer and mid-winter, to account for seasonal variability. The air pollution monitoring campaign was undertaken over two-week periods with a one-week break in between to allow for sample preparation, for a duration of nine consecutive months. An annual average concentration was estimated from the results of the two measurements for each sampling site by adjusting it with data from the Air Quality Monitoring Station of the eThekwini Municipality. At one additional site (reference site), the pollutants were measured using the same sample media for the full year to allow for the site-specific measurements to be temporally adjusted to the long-term annual average for the observation period [38]. An annual adjusted average prenatal PM_2.5,_ SO_2_ and NOx exposure ($\mu g/{m}^{3}$) was predicted, using a combined land use regression model based on pre-selected geographic predictors, such as land use types (area of industrial land use, open space land use, and the harbour), road length, topography, population, and housing density. The model developed accounted for 73% of the variance in ambient PM_2.5,_ SO_2_ and NOx measurements. No temporal adjustments were made. The parameter estimates were used to predict PM_2.5,_ SO_2_ and NOx exposure at the residential addresses of the study participants.

### Outcome variables

The three adverse birth outcomes examined in this study were the following: PB, LBW and SGA. All of the data were extracted from MACE data. Gestational age was assessed by obstetricians based on last menstruation or early ultrasound estimates. PB was coded as a dichotomous variable as infants were born before 37 completed weeks of gestation or not. Birthweight data were coded as a dichotomous variable, indicating LBW as a birthweight $\le$ 2,500 g (g). SGA was defined as $\le$ the 10.^th^ percentile for birth weight by gestational age [39, 40] across our sample and was categorised as a dichotomous variable. Birthweight (BW) measurement was obtained by trained nurses. Exploration of the data was performed using parallel coordinate plots, in order to examine trends of adverse birth outcomes across exposure to air pollution, and clinical factors. Details about parallel coordinate plots data visualization have been previously described [41].

### Prenatal risk factors

This study considered observed covariates as prenatal risk factors. These are exposure to ambient PM_2.5,_ SO_2_ and NOx pollution and clinical (gestational weight gain, BMI in the first trimester, and HIV status). Maternal socio-demographic status included demographic variables (maternal age, infant gender and low socio-economic status (unemployment, multiparous, low income), primary or less education), and low socio-economic housing). The perinatal health behavioural characteristics included alcohol use, smoking, and passive exposure to tobacco smoke during pregnancy. We also included walking and physical exercise as indicators of physical activity. Gestational weight gain was obtained as the difference in kilograms between the weight at the third and first trimesters and maternal BMI (in kg/m^2^) was calculated using first-trimester weight and height.

### Generalised Structural Equation Model (GSEM)

A structural equation model [42] is a multivariate statistical model that involves relationships among endogenous and exogenous latent variables, accounting for measurement error. It provides a general framework for modelling stochastic dependence that arises through cause-effect relationships between random variables. SEM minimises the effect of residual confounding in associations, especially in observational studies [43]. It allowed including variables with a mediating effect on the exposure and outcome variables. A SEM model is composed of two sub-models: a measurement model and a structural or causal model. In path diagrams of SEM, the ovals signify latent variables and observed variables are shown in rectangles. A structural model constitutes a directional chain system that describes the hypothetical causal relationship between the constructs of theoretical interest (latent variables) using path diagrams [44, 45]. The structural component of the model has the following mathematical form:

1 $${{\varvec{\eta}}}_{{\varvec{i}}}={\boldsymbol{\alpha }}_{{\varvec{\eta}}}+\mathbf{\rm B}{{\varvec{\eta}}}_{{\varvec{i}}}+{\varvec{\Gamma}}{{\varvec{\xi}}}_{{\varvec{i}}}+{{\varvec{\zeta}}}_{{\varvec{i}}}$$

where ${{\varvec{\eta}}}_{{\varvec{i}}}$ is a vector of latent endogenous variables for unit i, ${\boldsymbol{\alpha }}_{{\varvec{\eta}}}$ is a vector of intercept terms, $\mathbf{\rm B}$ is the matrix of coefficients giving the expected effects of the latent endogenous variables (${\varvec{\eta}})$ one each other, ${{\varvec{\xi}}}_{{\varvec{i}}}$ is a vector of latent exogenous variables, ${\varvec{\Gamma}}$ is a coefficient matrix giving the expected effect of latent exogenous variables (${\varvec{\xi}}$) on latent endogenous variables (${\varvec{\eta}})$, and ${{\varvec{\zeta}}}_{{\varvec{i}}}$ is the vector of disturbances. I = 1, …, n, E(${{\varvec{\zeta}}}_{{\varvec{i}}}$) = 0, COV(${{{\varvec{\xi}}}_{{\varvec{i}}}}^{\boldsymbol{^{\prime}}}$,${{\varvec{\zeta}}}_{{\varvec{i}}}$) = 0, and (I-$\mathbf{\rm B}$) is invertible.

A measurement model describes the relationships between latent variables and their manifest variables. The measurement model is represented as

2 $${{\varvec{y}}}_{{\varvec{i}}}={\boldsymbol{\alpha }}_{{\varvec{y}}}+{{\varvec{\Lambda}}}_{{\varvec{y}}}{{\varvec{\xi}}}_{{\varvec{i}}}+{{\varvec{\varepsilon}}}_{{\varvec{i}}} \mathrm{ and}$$

3 $${{\varvec{x}}}_{{\varvec{i}}}={\boldsymbol{\alpha }}_{{\varvec{x}}}+{{\varvec{\Lambda}}}_{{\varvec{x}}}{{\varvec{\eta}}}_{{\varvec{i}}}+{{\varvec{\delta}}}_{{\varvec{i}}}$$

where ${{\varvec{y}}}_{{\varvec{i}}}$ and ${{\varvec{x}}}_{{\varvec{i}}}$ are vectors of the observed indicators of ${{\varvec{\eta}}}_{{\varvec{i}}}$ and ${{\varvec{\xi}}}_{{\varvec{i}}}$, respectively, ${\boldsymbol{\alpha }}_{{\varvec{y}}}$ and ${\boldsymbol{\alpha }}_{{\varvec{x}}}$ are intercept vectors, ${{\varvec{\Lambda}}}_{{\varvec{y}}}$ and ${{\varvec{\Lambda}}}_{{\varvec{x}}}$ are matrices of factor loadings or regression coefficients giving the effect of the latent ${{\varvec{\eta}}}_{{\varvec{i}}}$ and ${{\varvec{\xi}}}_{{\varvec{i}}}$ on ${{\varvec{y}}}_{{\varvec{i}}}$ and ${{\varvec{x}}}_{{\varvec{i}}}$, respectively, and $\bf {{\varvec{\varepsilon}}}_{{\varvec{i}}}$ and ${{\varvec{\delta}}}_{{\varvec{i}}}$ are the unique factors of ${{\varvec{y}}}_{{\varvec{i}}}$ and ${{\varvec{x}}}_{{\varvec{i}}}$. We assume that the unique factors ($\bf {{\varvec{\varepsilon}}}_{{\varvec{i}}}$ and ${{\varvec{\delta}}}_{{\varvec{i}}}$) have expected values of zero, have covariance matrices of ${{\varvec{\Sigma}}}_{{\varvec{\varepsilon}}{\varvec{\varepsilon}}}$ and ${{\varvec{\Sigma}}}_{{\varvec{\delta}}{\varvec{\delta}}}$, respectively, and are uncorrelated with each other and with ${{\varvec{\zeta}}}_{{\varvec{i}}}$ and ${{\varvec{\xi}}}_{{\varvec{i}}}$.

GSEM is a more flexible modelling approach than SEM, similar to a generalised linear model (GLM), as a more flexible alternative to ordinary least squares regression. The GSEM allows responses of continuous or binary, ordinal, count, or multinomial variables. GSEMs represents a generalisation of SEMs by allowing the use of discrete variables and non-Gaussian distributions. They combine observed (or manifest) and latent variables representing unmeasured constructs. A GSEM can be defined as

$${\varvec{\eta}}={{\varvec{f}}}_{{\varvec{\eta}}}\left({\varvec{\eta}},{\varvec{\xi}},{\varvec{\zeta}}\right)$$

4 $${\varvec{x}}={{\varvec{f}}}_{{\varvec{x}}}\left({\varvec{\eta}},{\varvec{\delta}}\right)$$

$$\bf \bf {\varvec{y}}={{\varvec{f}}}_{{\varvec{y}}}\left({\varvec{\eta}},{\varvec{\varepsilon}}\right)$$

where *x* and *y* are vectors of manifest variables and ${\varvec{\eta}},{\varvec{\xi}},{\varvec{\zeta}}$ represent the latent variables, while **δ** and **ε** denote the error terms. The functions (${\varvec{f}}_{\varvec{\eta}}$ ${\varvec{f}}_{\varvec{x}}$, ${\varvec{f}}_{\varvec{y}}$) provide a general way to represent the connections between the variables within the parentheses to those on the left-hand side of each equation in Eq. 4.

The models were estimated by using the robust maximum likelihood approach with a method of mode-curvature adaptive Gauss–Hermite quadrature (MCAGH), which is superior in terms of accuracy to the nonadaptive methods.

The goodness of fit for each model was assessed with the Akaike information criterion (AIC) and the Bayesian information criterion (BIC). Lower AIC and BIC values indicate better model fit. AIC and BIC both balance model fit with parsimony, and each penalises based on the number of parameters. BIC imposes a larger penalty for complex models. As a result, AIC may overfit the model while BIC may underfit the model, but generally, they correspond closely with one another [46]. In the current data, we have tested the GSEM model using the Bernoulli distribution with a probit, logit and complementary log–log link functions. The fitted model with a complementary log–log link was found with lower AIC and BIC values (Table 1). Then, our final model was fitted with a Bernoulli distribution and complementary log–log link.

**Table 1:** Testing the Bernoulli distribution family under different link functions

Family/link	AIC	BIC
Probit	13,727.7	14,041.7
Logit	13,720.2	14,025.3
Cloglog	13,717.2	14,022.3

Indirect effects were calculated by multiplying the slope coefficients on each path. They were then summed to obtain the overall indirect effect of the variable. Total effects were calculated as a sum of the direct and indirect effects and reported in effect coefficients. These values were obtained using the nlcom command. We have used multiple imputation to address these missing points. All analyses were performed at a 5% significance level using STATA 15.0.

## Results

### Data Exploration

**Fig. 2:**
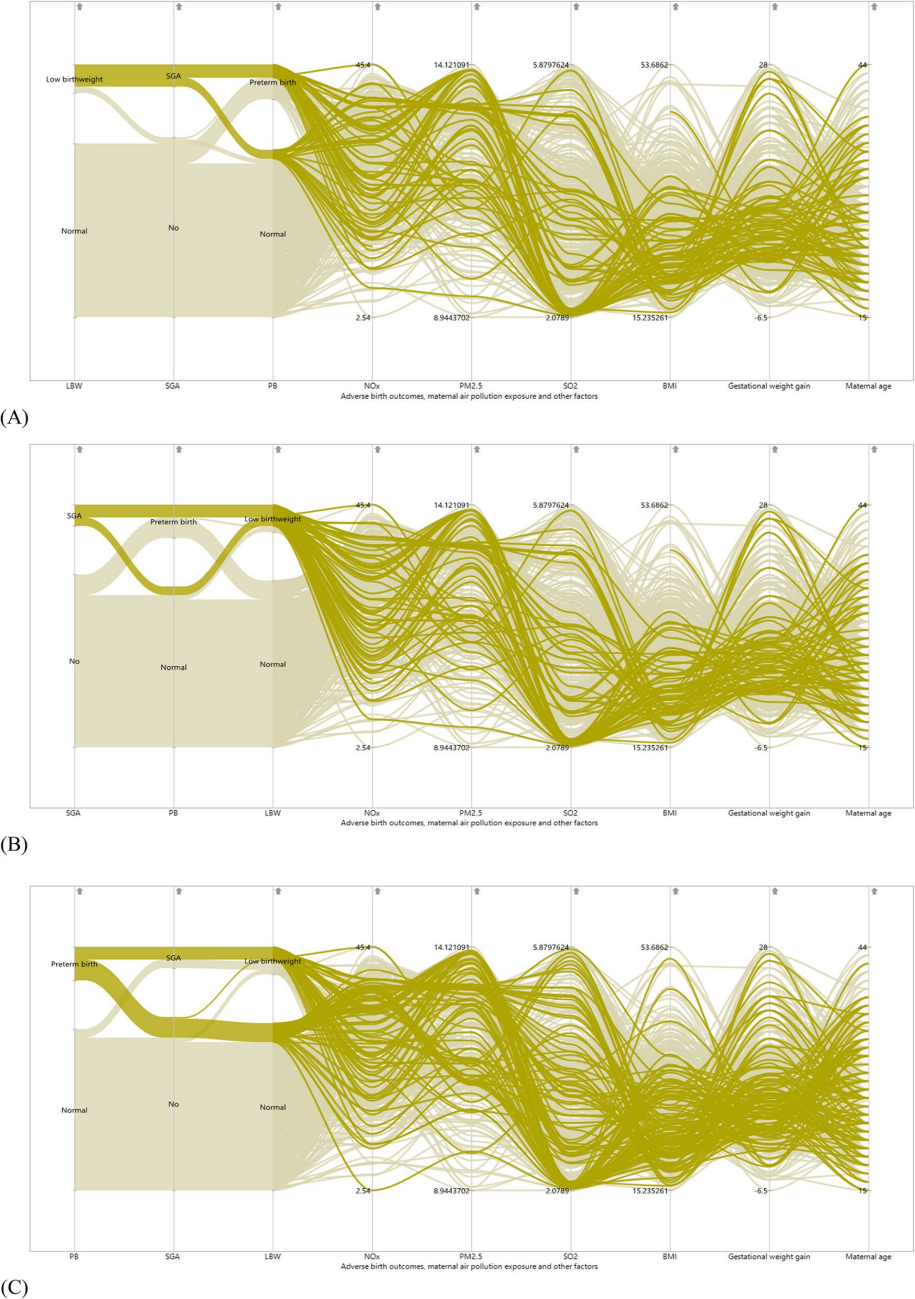
Parallel coordinates plot for trends of (**A**) low birthweight (**B**) small for gestational age (SGA) (**C**) preterm birth (PB) across maternal exposure to PM_2.5_, SO_2_ and NOx air pollution and other factors

**Fig. 3:**
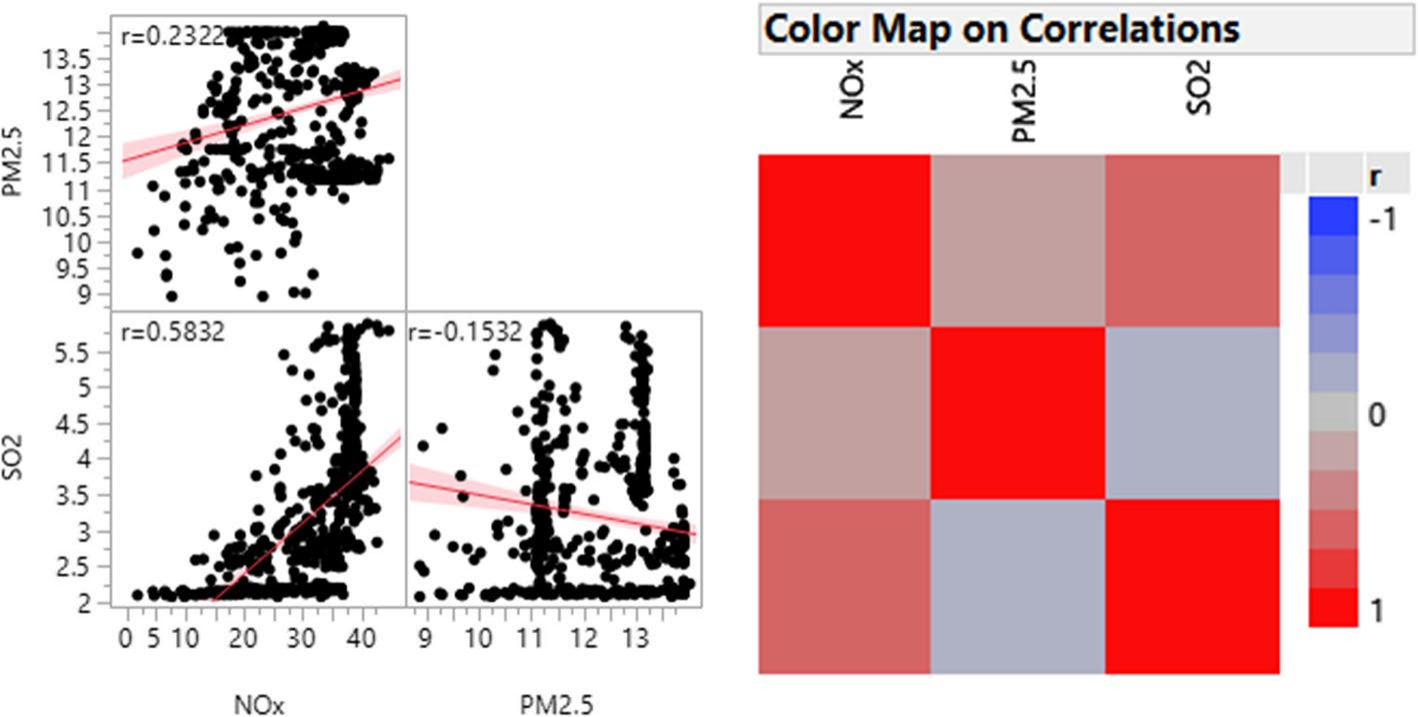
Correlation among measures of exposure to air pollution

The parallel coordinate plots revealed that mothers of infants with adverse birth outcomes LBW, SGA and PB tend to have average to higher prenatal exposure to PM_2.5_ (Fig. 2). For SO_2_ and NOx, similar trends of high range of variation from low to high were observed across different adverse birth outcomes. The parallel coordinate plots further displayed that infants with adverse birth outcomes were found to be born from mothers with lower BMI at first trimester (Fig. 2). Furthermore, both the scatter plot matrix and the colour map on correlations showed that exposure to NOx had a modest positive correlation with exposure to PM_2.5_ and SO_2_ pollution. On the other hand, PM_2.5_ is weakly and negatively correlated with exposure to SO_2_ (Fig. 3). This indicates the absence of multicollinearity among air pollution exposure measures. This all provokes the use of a comprehensive statistical model that considers the three adverse birth outcomes simultaneously to examine the adverse effect of air pollution and other adjusted factors.

The median annual air pollutant levels of PM_2.5,_ SO_2_ and NOx for individual women and the percentage of adverse birth outcomes by prenatal exposure factors for individual women and their newborns in the MACE birth cohort are shown in Table 2. The overall median level of exposure to PM_2.5,_ SO_2_ and NOx was 13.0 μg/m^3^ (range 8.9– 14.1 μg/m^3^), 2.8 μg/m^3^ (range 2.1 – 5.9 μg/m^3^), and 34.4 μg/m^3^ (range 2.5 – 45.4 μg/m^3^) respectively. The mean maternal age was 26 years (SD: 5.7 years). Of 656 infants in the birth cohort, 66.5% were from south Durban. The median level of exposure to PM_2.5_ was similar across all adverse birth outcomes while a higher median exposure level to NOx (34.6 μg/m^3^ (range 2.5 – 45.4 μg/m^3^)) was observed among mothers with preterm birth (Table 2).

**Table 2:** Percentage of adverse birth outcomes by prenatal exposure factors in the MACE Study (2013–2017)

Prenatal exposure factors	Percentage or Mean (SD)		
	LBW (*n* = 93)	SGA (*n* = 72)	PB (*n* = 113)
**Median annual exposure to air pollution (μg/m**^**3**^**)**			
**PM2.5**	13.2	13.2	13.2
**SO**_**2**_	2.6	2.2	2.7
**NOx**	32.6	29.2	34.6
**Behavioural factors**			
**Maternal smoking (Smoker)**	8.6	6.9	11.5
**Passive smoking (PSmoker)**	29.0	27.8	34.5
**Alcohol consumption**	9.7	9.7	8.0
**Low socio-economic factors**			
**Primary or less maternal education (PLEduc)**	3.2	2.8	5.3
**Maternal Unemployment (Unemp)**	18.3	20.8	21.2
**Low maternal annual income (LInc) (< US$2000)**	91.4	91.7	89.4
**Low socio-economic housing (LSEH)**	40.9	34.7	33.6
**Demographic factors**			
**Maternal age in years Mean (SD)**	25.7(6.5)	25.5(6.2)	25.9(5.7)
**Multiparous**	16.1	23.9	15.3
**Child gender (Female)**	45.2	44.4	50.4
**Clinical factors**			
**HIV status (Positive)**	34.4	34.7	36.3
**Syphilis (Positive)**	9.7	9.7	4.4
**BMI at first trimester (BMIT1) (kg/m**^**2**^**)**	25.5(6.6)	25.0(5.5)	25.8(6.3)
**Gestational weight gain (WeightGain) (kg)**	6.3(6.1)	6.6(6.1)	6.5 (6.0)
**Physical exercise (at least once in a week)**	45.2	44.4	44.2
**Residential location (South Durban)**	52.7	45.8	55.8

LSEH Flat, terraced flat, apartment building or Informa housing; Informal dwelling is a makeshift structure not erected according to approved architectural plans, for example, shacks or shanties in informal settlements or in backyards. [47]

**Fig. 4:**
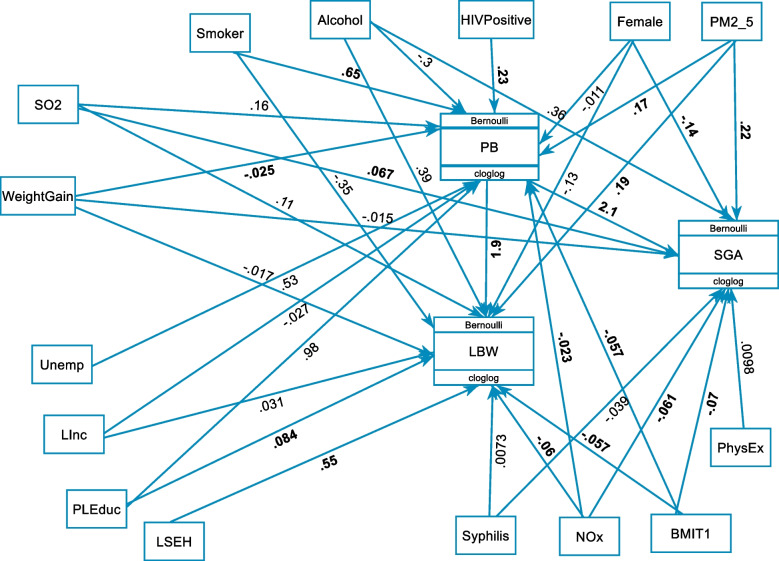
GSEM predicting adverse birth outcomes (LBW, SGA and PB) among infants from MACE birth cohort. Significant relationships bolded. (Passive smoking (PSmoker), Primary or less maternal education (PLEduc), Maternal Unemployment (Unemp), Low maternal annual income (LInc), Low socio-economic housing (LSEH), Physical exercise (PhyEx))

Figure 4 and Table 3 presents the final GSEM model containing both the structural and measurement components. The fitted model had a minimum AIC and BIC values compared to other competing models. It was found relatively parsimonious. The path diagram for the final model with all variables is given in Fig. 4. The single-headed arrows indicate causal effects and the associated parameter values show the coefficient estimates.

**Table 3:** Direct effects of GSEM predicting adverse birth outcomes (LBW, SGA and PB) among infants from MACE birth cohort

	**Adverse birth outcomes**		
	Low birthweight AOR (95% CI)	Small for gestational age AOR (95% CI)	Preterm birth AOR (95% CI)
**Prenatal exposure to air pollution**			
**PM2.5 (μg/m**^**3**^**)**	1.3(1.02, 1.42)*	1.2(1.21, 1.28)***	1.2(1.09, 1.29)***
**NOx (μg/m**^**3**^**)**	0.9 (0.92,0.95) **	0.9(0.93,0.95)**	0.98(0.96, 0.99)*
**SO**_**2**_** (μg/m**^**3**^**)**	1.1(0.94, 1..31)	1.1(1.01, 1..13)*	1.2(0.87, 1.59)
**Clinical factors**			
**Gestational weight gain (kg)**	0.9(0.94,1.03)	1.0(0.95, 1.03)	0.98(0.97, 0.99)***
**BMI T1 (kg/m**^**2**^**)**	0.9(0.92, 0.97)***	0.9(0.91, 0.95)***	0.94(0.93, 0.95)***
**Syphilis positive**	1.0(0.73, 1.39)	0.9(0.58, 1.59)	____
**HIV positive**	____	____	1.3(1.17, 1.35)***
**Behavioural factors**			
**Smoker**	0.7 (0.37, 1.36)	____	1.9(1.27, 2.89)**
**Alcohol use**	1.5(0.69, 3.19)	1.4(0.59, 3.48)	0.7(0.50, 1.09)
**Low socio-economic factors**			
**Primary or less education**	1.1(0.56, 2.12)	____	2.7(1.15, 6.17)*
**Low annual income (less than US$2000)**	1.0(0.47, 2.25)	____	1.0(0.55, 1.72)
**Low SE Housing (Informal or Flat, terraced flat, apartment building)**	1.7(1.61, 1.85)**	____	____
**Unemployment**	____	____	1.7(0.72, 3.97)
**Physical exercise (at least once in a week)**	____	0.01(-0.51, 0.02)	____
**Female gender**	0.87 (0.51, 1.51)	0.9(0.86, 0.89)***	1.0(0.86, 1.14)

95% confidence intervals based on standard errors clustered on residential location

*AOR* Adjusted Odds Ratio, *CI* Confidence interval

^***^P < 0.001

^**^p < 0.01

^*^P < 0.05

### Direct effects

Table 3 and Fig. 4 presents the coefficient estimates of direct effects of pathways between prenatal air pollution exposure and adverse birth outcomes from the fitted generalised structural equation model. Results showed that increased prenatal exposure to particulate matter PM_2.5_ increased the risk of LBW (AOR = 1.3, 95% CI:1.02–1.42). Prenatal exposure to SO_2_ was directly associated with SGA (AOR = 1.1, 95% CI:1.01–1.13). i.e. as exposure level to SO_2_ increases the probability of being born small for gestational age increases. The direct effects of prenatal exposure to NOx on adverse birth outcomes were significant, but not in the expected direction (Fig. 4 and Table 3). Our results suggest that infants born from smoker mother have a significantly increased risk of PB (AOR = 1.9, 95% CI: 1.27–2.89). Moreover, infants from HIV positive mothers had a higher tendency to be born preterm (AOR = 0.8, 95% CI:0.49–0.96).

The results also revealed that LBW (AOR = 0.9, 95% CI: 0.92–0.97), SGA (AOR = 0.9, 95% CI: 0.91–0.95) and PB (AOR = 0.94, 95% CI: 0.93–0.95) were negatively associated with increased BMI at first trimester. Increased gestational weight gain had associated with decreased odds of PB (AOR = 0.98, 95% CI:0.97 – 0.99). Among socio-economic variables, the results showed that the primary or less mother's education level had a negative significant association with PB (Fig. 4). Infants from mothers who had primary or less education have a significantly higher probability of being preterm (AOR = 2.7, 95% CI: 1.15–6.17). Our results also suggest that infants born from mother's living in lower socio-economic housing had associated with increased risk of LBW (AOR = 1.7, 95% CI: 1.61–1.85). Compared to male infants, females were less likely to be born with SGA (AOR = 0.9, 95% CI: 0.86–0.89) (Fig. 4 and Table 3).

### Indirect and total effects

Table 4 displays the indirect, and total effects of prenatal exposure factors with the adverse birth outcomes. We found that prenatal exposure to PM_2.5_ (AOR = 0.03, 95% CI: 0.02 – 0.04), and SO_2_ (AOR = 0.002, 95% CI: 0.01 – 0.02) and NOx (AOR = 0.001, 95% CI: 0.0003 – 0.002) exhibits indirect effect on LBW through PB. Furthermore, all of the three pollutants were associated with SGA indirectly through PB. However, these indirect effects were considerably of low effect sizes (Table 4). Infants from mothers with a higher level of exposure to PM_2.5_, SO_2_ and NOx are more likely to be LBW and SGA partly because of being preterm. Even if the direct effects were not in the expected direction, we observed a low-level indirect effect of prenatal exposure to NOx on LBW and SGA through PB (Table 4).

**Table 4:** Indirect and total effects of maternal prenatal exposure factors on adverse birth outcomes via preterm birth

Paths via PB	Coefficients (95%CI)	
	Indirect effect	Total effect
**NOx → LBW**	0.001 (0.0003, 0.002)**	1.91(1.49, 2.34) ***
**PM**_**2.5**_** → LBW**	0.03 (0.02, 0.04)***	1.94(1.53, 2.36) ***
**SO**_**2**_** → LBW**	0.002 (0.01, 0.02)***	1.93(1.50, 2.36) ***
**BMI T1 → LBW**	0.003(0.0.002, 0.005)***	1.91(1.49, 2.34) ***
**Weight gain → LBW**	0.0004(-0.0007, 0.002)	1.91(1.49, 2.34) ***
**NOx → SGA**	0.001 (0.0004, 0.002)**	2.1(1.45, 2.75) ***
**PM**_**2.5**_** → SGA**	0.04 (0.02, 0.05)***	2.1 (1.48, 2.80) ***
**SO**_**2**_** → SGA**	0.01 (-0.02, 0.04)***	2.1 (1.43, 2.78) ***
**BMI T1 → SGA**	0.004 (0.003, 0.005)***	2.1 (1.45, 2.75) ***
**Weight gain → SGA**	0.0004 (-0.0006, 0.001)	2.1 (1.45, 2.74) ***
**Female → SGA**	0.002 (-0.02, 0.02)	2.1 (1.43, 2.77) ***

^***^p-value < 0.001

^**^p-value < 0.01

Both the estimated direct effect of PM_2.5_ on LBW (AOR = 1.3, 95% CI:1.02–1.42) and indirect effect on through PB (AOR = 0.03, 95% CI: 0.02 – 0.04) are relatively higher, resulting a significant stronger positive total effect (total effect = 1.94, 95% CI:1.49, 2.34). The indirect effect points to the existence of a mediating effect of PB on the effects of prenatal exposure to air pollution on being born LBW. This suggests that preterm infants with increased prenatal exposure to air pollution were more likely to be born with LBW. Lastly, PB has a mediating effect on how BMI at first trimester affects LBW (indirect effect = 0.003, 95% CI:0.0.002, 0.005) and SGA (indirect effect = 0.003, 95% CI:0.0.002, 0.005) (Table 4).

## Discussion

Our study has demonstrated that the annual exposure to PM_2.5_ and SO_2_ air pollution constitute strong prenatal risk factor of adverse birth outcomes. The use of a novel statistical technique, GSEM showed that while the effects were mostly direct, the effect of air pollution through prenatal exposure to PM_2.5_ and SO_2_ on low birthweight and small for gestational age were mediated through preterm birth. This may be attributed to maternal exposure to PM_2.5_ increase throughout the entire pregnancy is related to an extra risk of preterm birth [48] and low birthweight may result from preterm birth [49]. The other possible reason may be including multiple measures of the mediating variable or construct, ceteris paribus, will be a better strategy for fully capturing percentage mediation [50].

Elevated prenatal maternal exposure to PM_2.5_ was positively associated with low birthweight, preterm birth and SGA. Echoing findings elsewhere [51, 52], our study confirms that PM_2.5_ has consistent adverse effects on adverse birth outcomes. Similar to our findings, a systematic review by Shah et al. [6] found that exposure to PM_2.5_ increases the risk of LBW, while.a study in Canada, indicated that a 10-μg/m^3^ increase in PM_2.5_ over the entire pregnancy was associated with small for gestational age [53]. Brauer et al. [54] found consistent associations between PM_2.5_ exposure and risk of preterm birth. Ambient PM_2.5_ exposure increased the risk of preterm birth increased by 3% for every 5 μg/m^3^ increase in PM_2.5_ average concentration in the entire pregnancy in one Chinese study [55]. A recent meta-analysis found maternal exposure to PM_2.5_ per IQR increment increase risk of PB throughout entire pregnancy [56].

Our finding suggests that a higher level of prenatal exposure to SO_2_ is associated with risk of small for gestational age. The concurs with a study in China, which showed a significant effect on adverse birth outcomes [54]. In this study, despite lower magnitude, we identified an indirect effect of exposure to PM_2.5_, and SO_2_ ambient air pollution on LBW and SGA. The mediation path contributing to this effect is through preterm birth. This suggests that preterm birth is an important mediator between prenatal exposure to ambient air pollution and adverse birth outcomes. While it may have a protective direct effect, NOx exposure has a positive indirect effect on LBW and SGA through preterm birth. This may be attributed to bias or residual confounding. However, these indirect association of NOx exposure with adverse birth outcomes are relatively weak in magnitude. Unlike this study by Brauer et al. found an association between exposure to NOx and LBW [54].

Mothers who are smokers were more likely to experience the adverse birth outcome of preterm birth compared to non-smokers. This is consistent with previous studies in the US, UK and Brazil that had shown the risk of PB is higher in smokers [57–59]. Our result is also in line with a recent systematic review and meta-analysis [60], in which smoking, was identified as a risk factor where smoking in pregnancy increased the risk of preterm birth. Similarly, Guan et al. found that smoking is a risk factor for preterm birth [61]. A recent study showed that women at greatest risk for PB are those with low socio-economic status, smoking [62].

This study used household-level air pollution estimates of exposure to pollutants NOx, PM_2.5_ and SO_2_, obtained through a land use regression model. Our work goes beyond previous findings by advancing a multivariate structural equation model to a more flexible generalised structural equation model, which allows effects of prenatal exposure to air pollution on, categorical responses, adverse birth outcomes. Another strength of this study was an adjustment for individual-level factors, such as maternal smoking status, weight gain, body mass index, syphilis and HIV status in addition to socio-demographic status, as compared to studies that utilise retrospective records, particularly from developing countries.

This study has a number of limitations. The main limitation of this study is the use of a single average exposure level of pollutants during the whole pregnancy. The effect of exposure to pollutants may have a differential effect on adverse birth outcomes at different trimesters. The LUR approach, the methodology used in several large epidemiological studies globally, including in birth cohorts, does not include a temporal component. In this study, only ambient air pollution exposure was available. Misclassification is also possible for the outcome variables, preterm birth and LBW. Misclassification of the mediator is important potential source of error which may impact on the exposure-outcome associations.

## Conclusion

In summary, this paper presented a Generalised structural equation model with a complementary log–log link that jointly explains adverse birth outcomes (low birthweight, SGA, and preterm birth), and prenatal exposure to ambient air pollution while accounting for socio-demographic, behavioural, physical activity and clinical risk factors. Our study revealed a consistent association of air pollution exposure to PM_2.5_ throughout pregnancy on increased risks of preterm birth, low birthweight and SGA.

Generalised structural equation modeling allowed investigation of the effect of prenatal air pollution exposures on adverse birth outcomes. Using this approach, we found that air pollution exposure had adverse effects on low birthweight and small for gestational age. This suggests that, while policies promoting reducing exposure levels of pollution will reduce preterm birth, its effect on reducing the likelihood of LBW and SGA. Furthermore, more research should also investigate whether the timing of environmental exposures during pregnancy (i.e., by trimester) is associated with adverse birth outcomes in our study setting.

## Supplementary Information

**Additional file 1:SupFigure 1.** Flowchart illustrating final numberof participant women used in the study from MACE birth cohort 

## Data Availability

The data that support the findings of this study are available from the MACE study but restrictions apply to the availability of these data, which were used under license for the current study, and so are not publicly available. Data are however available from the authors upon reasonable request and with permission from the MACE study.
