# Supplementary material for: Impact of ambient air pollution exposure during pregnancy on adverse birth outcomes: generalized structural equation modeling approach
Source: BMC Public Health. 2023 Jan 6;23:45. doi: 10.1186/s12889-022-14971-3 (PMC9824986; doi:10.1186/s12889-022-14971-3)
Supplement: Supplementary file 1 — Additional file 1:SupFigure 1. Flowchart illustrating final numberof participant women used in the study from MACE birth cohort [file 12889_2022_14971_MOESM1_ESM.docx]

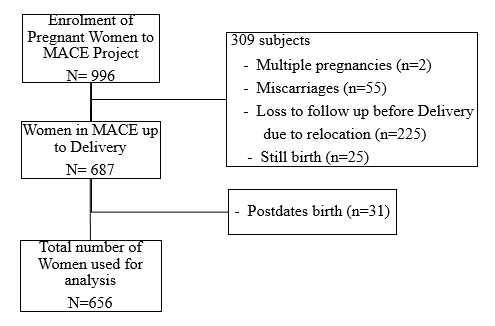


SupFigure 1. Flowchart illustrating final number of participant women used in the study from MACE birth cohort
